# Neural correlates of visual stimulus encoding and verbal working memory differ between cochlear implant users and normal‐hearing controls

**DOI:** 10.1111/ejn.15365

**Published:** 2021-07-09

**Authors:** Priyanka Prince, Brandon T. Paul, Joseph Chen, Trung Le, Vincent Lin, Andrew Dimitrijevic

**Affiliations:** ^1^ Evaluative Clinical Sciences Platform Sunnybrook Research Institute Toronto Ontario Canada; ^2^ Department of Physiology University of Toronto Toronto Ontario Canada; ^3^ Otolaryngology—Head and Neck Surgery Sunnybrook Health Sciences Centre Toronto Ontario Canada; ^4^ Faculty of Medicine, Otolaryngology—Head and Neck Surgery University of Toronto Toronto Ontario Canada; ^5^ Department of Psychology Ryerson University Toronto Ontario Canada

**Keywords:** cochlear implant, connectivity, hearing loss, neural oscillations, verbal working memory, visual processing

## Abstract

A common concern for individuals with severe‐to‐profound hearing loss fitted with cochlear implants (CIs) is difficulty following conversations in noisy environments. Recent work has suggested that these difficulties are related to individual differences in brain function, including verbal working memory and the degree of cross‐modal reorganization of auditory areas for visual processing. However, the neural basis for these relationships is not fully understood. Here, we investigated neural correlates of visual verbal working memory and sensory plasticity in 14 CI users and age‐matched normal‐hearing (NH) controls. While we recorded the high‐density electroencephalogram (EEG), participants completed a modified Sternberg visual working memory task where sets of letters and numbers were presented visually and then recalled at a later time. Results suggested that CI users had comparable behavioural working memory performance compared with NH. However, CI users had more pronounced neural activity during visual stimulus encoding, including stronger visual‐evoked activity in auditory and visual cortices, larger modulations of neural oscillations and increased frontotemporal connectivity. In contrast, during memory retention of the characters, CI users had descriptively weaker neural oscillations and significantly lower frontotemporal connectivity. We interpret the differences in neural correlates of visual stimulus processing in CI users through the lens of cross‐modal and intramodal plasticity.

## Graphical abstract

People with hearing loss who have a cochlear implant (CI) have altered neural processing when engaged in a visual working memory task. During the visual stimulus encoding, CI users had greater visual and auditory cortex activation compared with normal‐hearing controls. Greater connectivity between temporal and frontal areas was observed for CI users compared with normal‐hearing controls.

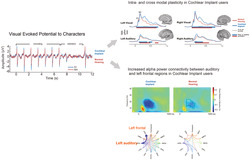

**Abbreviations**
BESA: Brain Electrical Source Analysis
CI: cochlear implant
EEG: electroencephalogram
ERD: event‐related desynchronization
ERS: event‐related synchronization
GC: Granger causality
HI‐MoCA: Hearing‐Impaired Montreal Cognitive Assessment
HINT: Hearing in Noise Test
LIFG: left inferior frontal gyrus
LSTG: left superior temporal gyrus
MSBF: multiple source beamformer
NH: normal hearing
REB: Research Ethics Board
ROI: region of interest
SNR: signal‐to‐noise ratio
TFR: time–frequency representation
TSE: temporal spectral evolution
VEP: visual‐evoked potential

## 1 INTRODUCTION

In the context of social settings, successful speech perception relies not only on the characteristics of auditory signals and the level of background noise but also on the visual speech cues that influence neural representations of auditory information (Hirst et al., 2018; Sumby & Pollack, 1954). Visual speech is especially important for hard‐of‐hearing individuals for whom auditory signals are degraded (Schorr et al., 2005; Stropahl et al., 2017). An abundance of human and animal research has suggested that a reduction or absence of auditory input over time leads to forms of cross‐modal plasticity, such as the reorganization of auditory brain areas for visual processing (Land et al., 2016; Lomber et al., 2010; Merabet & Pascual‐Leone, 2010). The cochlear implant (CI) population, due to prolonged periods of deafness or near deafness, present an opportunity to conduct a natural experiment to study cross‐modal plasticity and compensatory visual behaviours under conditions where hearing is restored after auditory deprivation.

Neuroimaging studies have broadly observed cross‐modal plasticity in deaf humans and CI users (Finney et al., 2001, 2003; Rouger et al., 2012; Stropahl et al., 2017). Song et al. (2015) additionally found greater visual activation for audiovisual speech in CI users, suggesting plastic effects on multimodal perception, as well as a weaker ‘bottom‐up’ drive to posterior superior temporal sulcus during audio‐only conditions. This could suggest an incomplete reversal of cross‐modal plasticity after hearing is restored (Rouger et al., 2012) or that auditory reorganization is maintained by a continued reliance on visual input.

Although cross‐modal plasticity may be considered an adaptive phenomenon, greater degrees of auditory reorganization appear to impede performance in speech perception tasks in CI users (Buckley & Tobey, 2011; Doucet et al., 2006; Merabet & Pascual‐Leone, 2010; Sandmann et al., 2012; Schierholz et al., 2015; but see Anderson et al., 2017; Land et al., 2016). Deafness‐related visual plasticity in auditory cortex may help to explain persistent speech listening problems that deaf individuals face after receiving a CI, and measurement may be important to understand how plasticity regresses once the auditory system is reafferented (Rouger et al., 2012). In contrast, stronger intramodal plasticity in the visual system is associated with better CI outcomes (Doucet et al., 2006; Strelnikov et al., 2013). These findings suggest that deafness‐related plasticity in sensory systems is not necessarily deleterious, but rather auditory remodelling per se might disadvantage CI rehabilitation.

While visual plasticity is one factor that can influence speech perception in CI users, the role of cognition also must be considered. In general, the consequences of long‐term auditory deprivation and reafferentation on cognitive function for speech understanding are not firmly established but are a topic of considerable focus (Peelle et al., 2011). From the perspective of auditory processing, working memory plays a critical role when auditory input is degraded by environmental noise or use of a hearing prosthesis. One view is that degraded auditory information must be maintained in working memory while it is matched to phonological representations stored in long‐term memory (Mattys et al., 2012; Ohlenforst et al., 2017; Rönnberg et al., 2010). Accordingly, CI users exhibit delays in the identification of consonants, individual words and the final words of sentences in noise (Finke et al., 2017; Moradi et al., 2014) which suggests an increased engagement of working memory systems.

Because speech representations are multimodal, the role of visual cognition is of interest in light of cross‐modal visual plasticity and putative changes to auditory working memory in CI users. Aside from visual cues taken from mouth, face and manual gestures, individuals with hearing loss also leverage verbal information presented through text (e.g., closed captioning) to facilitate speech perception (Gordon‐Salant & Callahan, 2009). Verbal working memory for both auditory signals and visual text are expected to engage the phonological loop, in which verbal information can be stored using active rehearsal or subvocalization of inner speech. (Baddeley, 2000; Baddeley et al., 1975). Further, the neural correlates of verbal working memory appear to overlap in prefrontal cortex, intraparietal sulcus and supramarginal gyrus (Crottaz‐Herbette et al., 2004). Behavioural evidence for problems with verbal working memory have mainly been shown in prelingually deafened children with CIs. Children with CIs have slower speaking rates (Burkholder & Pisoni, 2003), suggestive of slower covert verbal rehearsal and shorter memory spans (Baddeley et al., 1975; Hitch et al., 1989; Hulme & Tordoff, 1989; Kail & Park, 1994; Schweickert et al., 1990) and poorer performance on visual digit span tests (AuBuchon et al., 2015). They also have longer interword pause durations during digit span recall, indicating that they were slower at scanning items in their short‐term memory (Burkholder & Pisoni, 2003; Clifton & Tash, 1973; Sternberg, 1980). Data from predominantly postlingually deafened adults has however been mixed. One study found that adult CI users were marginally worse on a working memory task involving the reproduction of the order of animal pictures (which could be rehearsed verbally; Moberly et al., 2016), while other studies have found no difference in digit span or object span compared with normal‐hearing (NH) controls (Moberly, Pisoni, et al., 2017). Thus, the effect of profound hearing loss treated with a CI on working memory and its neural correlates are unclear.

One form of neural activity associated with working memory is scalp‐recorded neural oscillations. Modulations of alpha (8–12 Hz) and beta (13–30 Hz) synchrony measured using magneto/electroencephalography (M/EEG), for instance, represent gating of sensory information operating to protect or apply information retained in working memory (Bonnefond & Jensen, 2012; Jensen, 2002; Jensen & Mazaheri, 2010; Klimesch et al., 2007; Sauseng et al., 2005; Tuladhar et al., 2007). Alpha oscillations appear to be particularly sensitive to hearing loss in working memory paradigms. In an auditory speech‐based task, Petersen et al. (2015) found that alpha power increased with memory load in individuals with milder hearing loss, but those with severe hearing loss appeared to hit a ‘cognitive limit’, with alpha power significantly attenuated when task demands were high. This finding concurs with several reports indicating that strain on cognitive resources for auditory perception leaves fewer available for cognitive processing (Arlinger et al., 2009; Lunner et al., 2009; Pichora‐Fuller & Singh, 2006; Pichora‐Fuller et al., 2016; Rudner et al., 2011). Whether or not similar effects extend to visual verbal working memory in CI users, either speaking to a general change to memory function and neural resource management as a consequence of hearing deprivation or to prolonged use of visual cues per se, remains to be observed. These factors may also explain CI users' auditory‐only speech performance.

Pursuant to these questions, here, we measured visual encoding and visual working memory in CI users and compared them with controls matched precisely in age. To draw a clean line separating visual encoding and memory processes, we opted to record the EEG during a modified Sternberg working memory task (Obleser et al., 2012) in which stimuli were visual characters presented sequentially (visual stimulus encoding) followed by a period where participants held the information in working memory (retention). We tested the following hypotheses: (1) behavioural visual working memory performance in CI users is significantly different from controls, (2) evoked responses to visual information are larger in CI users compared with controls, as evidence of cross‐modal plasticity, (3) neural alpha oscillations related to working memory performance are significantly different from controls, (4) neural variables that were significantly different between groups uniquely explain variability in behavioural performance (i.e., brain–behaviour correlations) and (5) neural correlates of visual working memory and visual encoding explain individual differences in clinical speech‐in‐noise scores in CI users.

## 2 METHODS

### 2.1 Participants

Demographic information for all participants is given in Table 1. Fourteen CI users were recruited from the patient population in the Department of Otolaryngology at Sunnybrook Health Sciences Centre. CI users were aged between 18 and 77 years (*M* = 50.7, SD = 19.8) and included six males and eight females with no underlying neurological conditions. This group consists of one bilateral CI user and 13 unilateral CI users, including four unilateral CI users who used a hearing aid on their contralateral ear. As part of their standard clinical testing, speech perception‐in‐noise scores were measured using the AzBio test (Spahr & Dorman, 2005) and the Hearing in Noise Test (HINT; Nilsson et al., 1994) administered 1 year or more after activation of the CI at a signal‐to‐noise ratio (SNR) of +5 dB and were used for correlational analysis. For two out of the 14 participants, their HINT scores were used. For each individual, their last speech‐in‐noise test score was used for correlational analysis, which was at most 2 years before the EEG test was done. Despite the difference between HINT and AzBio, studies have reported similar performance outcomes in quiet and in noise as well as similar median times at which the plateau in performance had occurred (Massa & Ruckenstein, 2014; Sevier et al., 2019). One participant's speech‐in‐noise score was not available, and they were not included in analyses involving speech‐in‐noise tests. Duration of deafness before implantation was defined as the participant's subjective report of deafness onset subtracted from date of implantation. In addition, 14 age‐matched controls (age range 18–72, *M* = 49.9, SD = 19.2; seven males and seven females), recruited through local databases and online social media groups in the Toronto, Canada, area, also participated in the study and served as the control group.

**TABLE 1:** Demographic of CI and NH participants

CI participants	Age	Gender	Speech perception‐in‐noise score	Condition	Duration of CI use (years)	Duration of deafness (before implantation in years)	Aetiology
1	18	Male	83	Bilateral CI	16.6	1.4	Congenital: unknown
2	25	Female	40	Right CI only	21.7	3.3	Homozygous recessive GJB2 gene mutation
3	25	Female	N/A	Left CI only	15	10	Congenital: unknown
4	28	Male	68	Left CI only	1.3	21.7	Hereditary: unknown
5	36	Female	87	Left CI only	1.3	32.7	Usher syndrome
6	57	Female	75	Left only (right plugged)	2.6	15.4	Hereditary: unknown
7	57	Male	76	Left CI only	1.8	25.2	Bilateral Meniere's
8	60	Female	30	Left CI only	0.9	21.1	Unknown
9	62	Male	48 (HINT)	Left CI + right HA	1.9	7.1	Unknown
10	65	Female	0 (HINT)	Left CI + right HA	2.3	57.7	Early childhood: unknown
11	65	Male	59	Left CI only	1.6	33.4	Progressive SNHL: noise induced
12	66	Female	75	Right CI only	1.2	8.8	Hereditary: progressive bilateral cochleovestibular loss
13	69	Male	0	Left CI + right HA	1.6	7.4	Meningitis
14	77	Female	22	Right CI + left HA	4.8	26.2	Progressive SNHL: noise induced

NH participants	Age	Gender					
1	18	Male
2	23	Female
3	23	Female
4	30	Male
5	36	Female
6	57	Male
7	59	Male
8	60	Male
9	61	Female
10	63	Female
11	63	Female
12	65	Female
13	68	Male
14	72	Male

*Note:* For each group, the individuals are numbered 1 to 14 from youngest to oldest, and their corresponding age and gender are recorded, respectively, in the columns to the right. The CI participants table includes five extra columns listing the outcomes of their speech perception tests (AzBio or HINT), their condition (bilateral, unilateral and/or if a hearing aid [HA] is used, specifying left or right ear lateralization), duration of CI use in years, duration of deafness before implantation and aetiology of their condition.

Abbreviations: CI, cochlear implant; HINT, Hearing In Noise Test; NH, normal hearing; SNHL, sensorineural hearing loss.

All participants had normal visual acuity as tested by the Freiburg Visual Acuity & Contrast Test (FrACT; Bach, 2006). Younger participants in the control group had NH thresholds in left and right ears (below 20‐dB hearing level (HL)) when tested at octave steps between 250 and 8000 Hz. Participants 57 years and older had NH thresholds in the lower frequencies (250–2000 Hz) but had threshold shifts to a maximum of 80 dB HL at frequencies above 4000 Hz. These levels are consistent with normal age‐related threshold shifts (Baraldi et al., 2007).

All participants provided written and informed consent for the study procedures, which were conducted in accordance with the Research Ethics Board (REB) at Sunnybrook Health Sciences Centre. The approved protocol was in agreement with the Declaration of Helsinki. Participants were monetarily compensated for their participation and were provided full reimbursement for parking fees at the hospital campus.

### 2.2 Working memory task

#### 2.2.1 Modified Sternberg task stimuli and materials

The primary task given to all participants was a modified Sternberg task, wherein sequences of seven random letters and numbers were individually presented, and after a short period of holding those items in working memory, participants reported if a target character was in the originally presented set. Individual stimuli were the eight most common letters in the English language (Lewand, 2001), including E, T, A, N, S, H, R and D, as well as the numbers 2 through 9. The letters ‘I’ and ‘O’ were excluded because they can be confused for the numbers ‘1’ and ‘0’ (zero), which were also excluded. All characters were shown as white characters centred on a black background and were presented on a computer monitor. The height of all characters was 5 cm on the monitor. Participants completed the task in a seated position approximately 140 cm away from the computer monitor, as measured from the nasion to the screen. Testing was completed within a sound‐attenuated and electrically shielded booth.

#### 2.2.2 Experimental procedure

**FIGURE 1:**
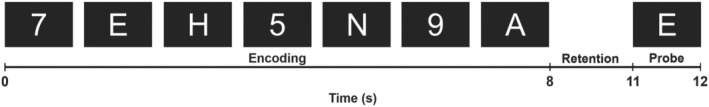
Visual working memory task paradigm. This entails the encoding phase, in which seven random characters (numbers or letters) appear on a computer screen 1 s apart from each other; the retention phase, a 3‐s interval to hold information; and lastly, the retrieval phase, a probe is shown to which the participants answer yes or no. One trial lasts 12 s in total

The trial structure of the task is shown in Figure 1. First, a fixation cross was placed on the computer monitor for 1 s. After, seven characters from the stimulus set (randomly chosen on each trial with no repeating characters) appeared sequentially on the computer screen. Characters were presented for a duration of 1 s before the next character appeared 0.1 s later. Herein, the time during which stimuli were presented is referred to as the encoding period. After the presentation of the final character, a black screen was presented for 3 s. During this time, participants were instructed to hold the entire seven‐character set in memory. This time interval is referred to as the retention period. Finally, a probe character was presented at the end of the retention period. Participants indicated by key press on a keyboard if the probe character was in the list of items shown during the encoding period. The probe character had a 50% probability of being in the original character set.

Participants completed eight blocks of 25 trials each, totalling 200 trials. CI users completed all trials without their CI processor or hearing aid on. After each block, the participants were verbally asked to rate their ‘effort’ and the ‘difficulty’ of the task. This was described to them as how much effort they put into completing the task and how difficult the task was, respectively. Participants were asked to rate both ‘effort’ and ‘difficulty’ on a 0 to 10 numerical rating scale. For effort, ‘0’ meant no effort, and ‘10’ indicated the most effort possible. For difficulty, ‘0’ indicated not difficult at all, and ‘10’ represented extremely difficult. Behavioural working memory performance was calculated as a percentage of trials in which the participants correctly classified the probe as being within or absent from the presented character set.

### 2.3 EEG recording and preprocessing

The EEG was recorded using CURRY software (Compumedics Ltd, Victoria, Australia) and was sampled at 2 kHz using a NeuroScan SynAmps II amplifier (Compumedics Ltd, Victoria, Australia) from 64 equidistant sensors on an ActiCAP (BrainProducts, Gilching, Germany) cap and referenced online to the vertex electrode. The equidistant layout covers a larger area than a standard 10–20 system, in order to improve source localization estimates (Dimitrijevic et al., 2017, 2019). The 3D surface electrode positions for each participant were digitally mapped using a Polhemus Patriot (Polhemus, Colchester, VT, USA).

Using Brain Vision Analyzer software (Brain Products, Gilching, Germany), raw EEG data were first filtered from 0.1 to 40 Hz through a second‐order Butterworth filter and then downsampled to 250 Hz. Continuous data were then subjected to independent component analysis (ICA) to identify myogenic artefacts, (e.g., eye blinks and eye movements) and other contaminants (e.g., intermittent faulty electrodes). Artefactual noise was confirmed by visual inspection, and the corresponding independent component weights were set to zero before the EEG was reconstructed. Between five and eight artefacts were removed per participant. Noisy channels were replaced by derived estimates from neighbouring sensors using spline interpolation. After, continuous EEG data were exported into Brain Electrical Source Analysis (BESA) software (BESA, GmbH, Germany) for analyses.

### 2.4 Neural activity during stimulus encoding

#### 2.4.1 Sensor‐level analysis of VEPs

Visual‐evoked potentials (VEPs) were examined in two ways. First, *trial‐averaged* VEPs elicited by the presentation of the seven visual characters and the probe were segmented into 13‐s epochs, from 1 s before the start of each trial (trials commenced at the onset of the first character) to 12 s (0.004 s after the offset of the probe), and averaged. Second, *event‐averaged* VEPs (averaged across the seven characters and across trials) were obtained by segmenting 1.5‐s epochs around the onset of each character, spanning 0.2 s before the onset of each character to 1 s after onset. EEG data for each VEP were re‐referenced to the scalp average and baseline corrected to the −0.5‐ to 0‐s interval. Trials containing noisy artefacts, not corrected for by ICA, were removed by visually inspecting and removing trials with any channel exceeding 120 μV. The resulting individual data files were exported from BESA and imported using the Fieldtrip toolbox (Oostenveld et al., 2011) in MATLAB (2019a, The Mathworks, Inc., Natick, MA, United States).

EEG sensors for analysis of VEPs were chosen based on the well‐established observation that VEPs reach their maxima in occipital channels (Kothari et al., 2016). This was corroborated by an inspection of grand average responses across participant groups, and eight channels across the occipital region were chosen for analysis and visualization of VEPs. Two VEPs were analysed; first, the positive‐going P1 response occurring near or just before 100‐ms poststimulus onset and the N1 response, occurring approximately at 130‐ms poststimulus onset (Odom et al., 2004). For each participant, voltages were averaged across a 20‐ms window based on the peak of the grand average responses for both groups.

#### 2.4.2 Source analysis of VEPs

Sources of VEPs were computed using standardized low‐resolution electromagnetic tomography (sLORETA) modelling (Palmero‐Soler et al., 2007; Pascual‐Marqui, 2002) using the default settings in Brainstorm (Tadel et al., 2011). A boundary element model (BEM) head model was created in the OpenMEEG plugin in Brainstorm. Each sLORETA map was used to extract the absolute values of the source time series (aka ‘scouts’) in predefined regions of interest (ROIs) of bilateral auditory and occipital cortices based on the Desikan–Killiany atlas (Desikan et al., 2006) following regions suggested by Stropahl et al. (2017). All four ROIs had maximal activations during the N1 time window. Group comparisons were performed on the ROIs activation on a 40‐ms time window centred on the N1 peak.

#### 2.4.3 Time–frequency decomposition and source analysis of VEPs

To obtain an average time–frequency representation (TFR) of VEPs, continuous EEG data were segmented into 1.2‐s epochs, from 0.2 s before the onset of a character (indicated by a visual trigger) to 1 s after the onset. In BESA, temporal spectral evolution (TSE; Vázquez et al., 2001) was used to compute TFRs with a frequency resolution of 2 Hz from 4 to 50 Hz and a temporal resolution of 25 ms. TFRs for the encoding period were measured using the same channel combinations described above for analysis of VEPs. The second analysis used the Multiple Source Beamformer (MSBF), a BESA implementation of the linearly constrained minimum variance (LCMV) vector beamformer that is suitable for TFRs. MSBF was applied to time and frequency windows of interest that were determined from the grand average TFR in each group.

#### 2.4.4 Connectivity analysis during visual stimulus encoding

**FIGURE 2:**
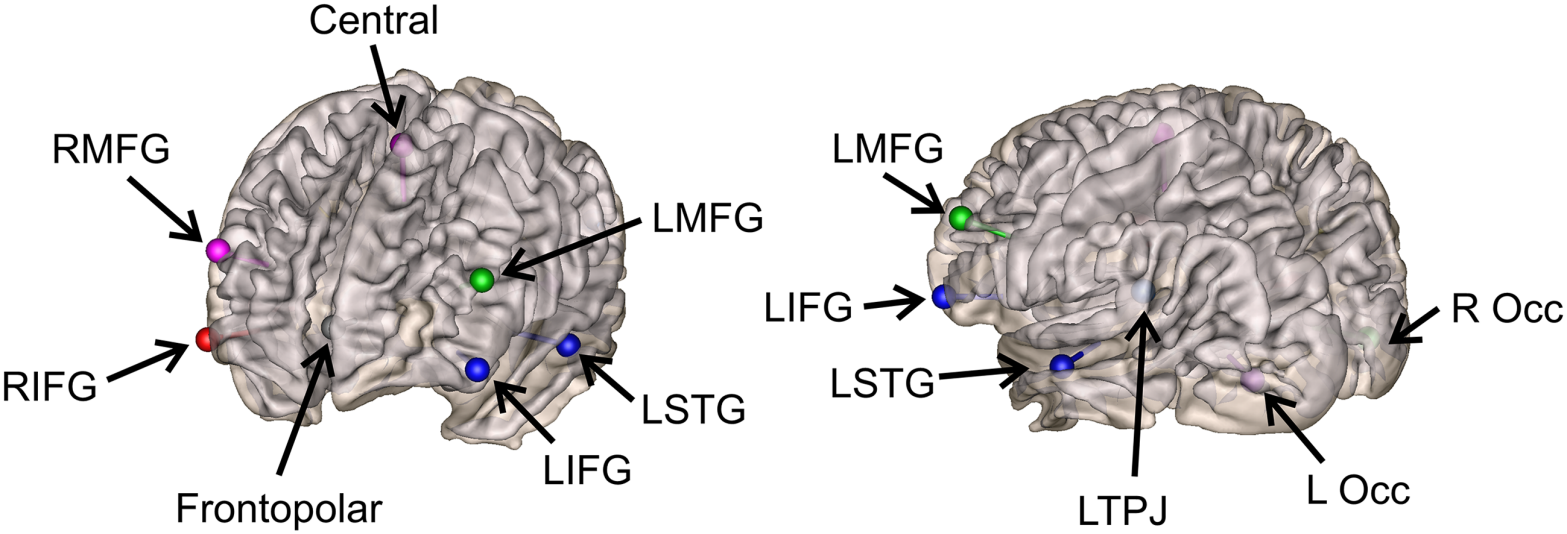
Location of regions of interest (ROI) dipoles. ROIs are presented as dipoles in their respective locations labelled by their abbreviations and approximate Talairach coordinates: left and right inferior frontal gyri (LIFG and RIFG; ±42, 39, 1), left and right middle frontal gyrus (LMFG and RMFG; ±43, 41 26), left and right temporal parietal junction (LTPJ and RTPJ; ±51, −48, 26), left and right superior temporal gyri (LSTG and RSTG; ±53, −23, −8), left and right occipital cortices (L_Occ and R_Occ; ±26, −82, −3), frontopolar region (0, 49, 15) and central region (0, −14, 53)

To investigate how brain sources are functionally connected, Granger–Geweke‐based connectivity analysis, a multivariate autoregressive model, (Geweke, 1984) was applied to the TFRs separately in the encoding and retention period using BESA Connectivity 1.0 to calculate connectivity between chosen ROI. The recorded sensor‐level data were translated to source space using the BESA montage ‘Ventral Attention with Noise Sources’ containing 12 ROIs. This was chosen based on sources associated with visual working memory and a pattern of significant source activation data in this study (mainly focusing on components of the occipital, frontal and temporal regions). The ROIs are left and right inferior frontal gyri (LIFG and RIFG), middle frontal gyrus (LMFG and RMFG), temporal parietal junction (LTPJ and RTPJ), superior temporal gyri (LSTG and RSTG), occipital cortices (L_Occ and R_Occ), frontopolar region and central region (Figure 2). For each individual, source waveform data were exported from BESA Research to BESA Connectivity at which point time–frequency decomposition was computed through the Complex Demodulation approach (Papp & Ktonas, 1977). Sampling was set to the settings utilized for time–frequency analysis described above with a 5 and 50 Hz frequency cut‐off. Then, connectivity analysis by Granger–Geweke causality in the frequency domain was applied through non‐parametric means (100 iterations, 0.0001 tolerance and 0.01 regularization), resulting in a 12 × 12 connectivity matrix that was used for statistical analysis. A grand average connectivity matrix was calculated in MATLAB, where the matrices for each individual within a group are averaged and then plotted as a web of connections (Kassebaum, 2020).

### 2.5 Neural activity during working memory retention period

In BESA, TFRs for the −1‐ to 13‐s epoch during the retention period were computed using TSE, as was done for the encoding period. MSBF was used for source reconstruction for alpha oscillations during the retention period (8 to 10.5 s). Granger causality (GC) was also calculated on this time window between the 12 nodes specified in the analysis for the encoding period.

### 2.6 Statistical analysis

Depending on the analysis, statistical tests were performed using R (R Development Core Team, 2019), BESA Statistics 2.0 or Brainstorm. Using built‐in functions in R, unpaired‐sample *t* tests were used to compare behavioural performance, response times, effort and difficulty scores, and Spearman correlation tests analysed relationships between behavioural variables. Effect sizes for *t* tests are expressed by *η* ^2^ (note that this is analogous to *r* ^2^ which describes the variance explained by the group difference, but to stay consistent with effect sizes reported for analysis of variance below).

Sensor‐level VEPs and source activations of VEPs were analysed using unpaired *t* tests. For source activations, unpaired *t* tests between NH and CI groups were computed in Brainstorm and were corrected for repeated measures using cluster analysis on ROI time series waveforms. Paired *t* tests compared changes in activation magnitude from baseline within each group. As a follow‐up, source activations were analysed in a 2 × 2 × 2 mixed ANOVA (*afex* package in R) comparing within‐subjects factors of hemisphere (left vs. right) and cortex (auditory vs. visual) and a between‐subjects factor of group (CI vs. NH). Post hoc comparisons were completed using the *emmeans* package and were corrected for false discovery rate (Benjamini & Hochberg, 1995). Results are reported alongside *η* ^2^ to express effect size.

To compare visual‐evoked oscillations during the encoding and retention period, as well as GC values, cluster‐based permutation tests with Monte Carlo approximation (5000 permutations) were implemented in both BESA Statistics 2.0 and Brainstorm using the FieldTrip plugin.

Relationships between behavioural data, including durations of deafness before implantation, duration of implantation and speech‐in‐noise scores and neural variables, were analysed using Spearman correlations. The 14 age‐matched controls did not have speech perception‐in‐noise scores, and therefore, correlational analyses with speech perception were only performed on neural and behavioural data from the CI group.

All *t* tests and Spearman correlations were two tailed, and the alpha criterion for type I error was set at 0.05.

## 3 RESULTS

### 3.1 Behavioural results

**FIGURE 3:**
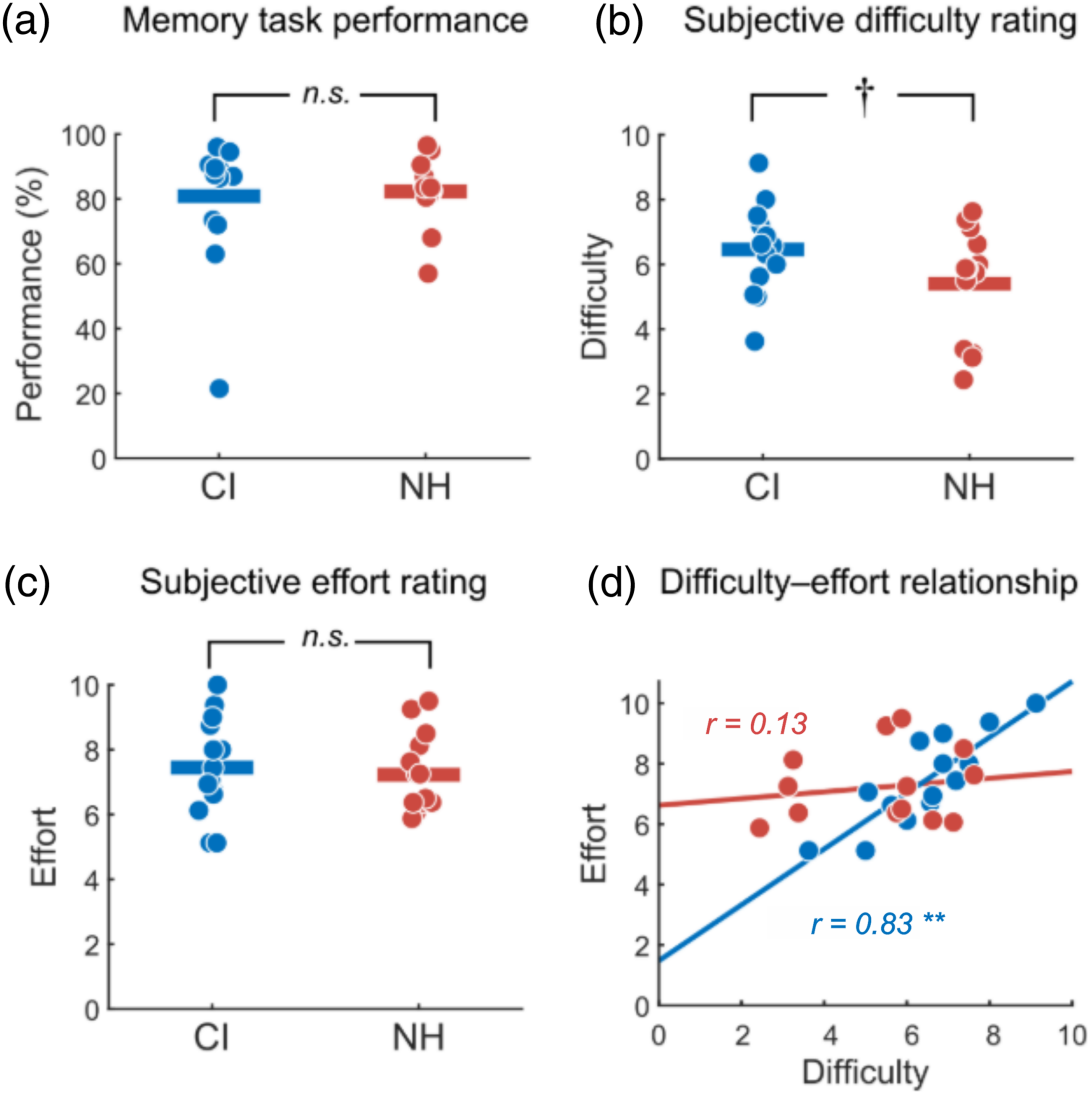
Behavioural measures for cochlear implant (CI) and normal‐hearing (NH) groups. (a) Working memory task performance. (b) Subjectively rated difficulty of the task. (c) Subjectively rated effort given during the task. For (a)–(d), individual circles represent individual subjects in each group; horizontal lines represent group means. (d) Individual differences in the relationship between subjectively rated task difficulty and effort. Lines indicate least squares fit for both CI and NH groups. ^**^ *p* < 0.001, ^†^ *p* < 0.10; n.s. = not significant

Figure 3 plots working memory task performance, subjectively rated task difficulty and subjectively rated effort ratings of all participants in each group. Respectively, averages and standard deviations of these values for CI users were 81% (0.20), 6.46 (1.37) and 7.45 (1.50). For NH, averages and standard deviations were 83% (0.10), 5.41 (1.69) and 7.23 (1.21). We note here that one participant in the CI group appeared to perform the task well below chance, at 20% accuracy. A plausible explanation is that this participant confused the ‘yes/no’ configuration when responding by keyboard keypress. Omission of this data point from behavioural analysis did not change outcomes of the statistical tests.

Descriptively, CI users and NH controls were comparable in performance on the task. The CI users found the task more difficult and reported higher effort. Statistically, task behaviour and effort and demand ratings in CI users were not significantly different from controls; however, the increase in difficulty for CI users over the NH group was close to significance (*t*(26) = 1.80, *p* = 0.08, *η* ^2^ = 0.11). No significant correlations were found between behavioural and self‐report measures with the exception of a positive correlation between difficulty and effort scores in CI users (*ρ* = 0.83, *p* = 0.0002). This relationship was not significant in NH controls, (*ρ* = 0.13, *p* = 0.65), and the correlation between effort and difficulty was significantly different between the NH and CI groups (Fisher's *r*‐to‐*z* transformation = 2.48, *p* = 0.01).

### 3.2 Visual encoding results

#### 3.2.1 Differences in VEPs between CI users and NH controls

**FIGURE 4:**
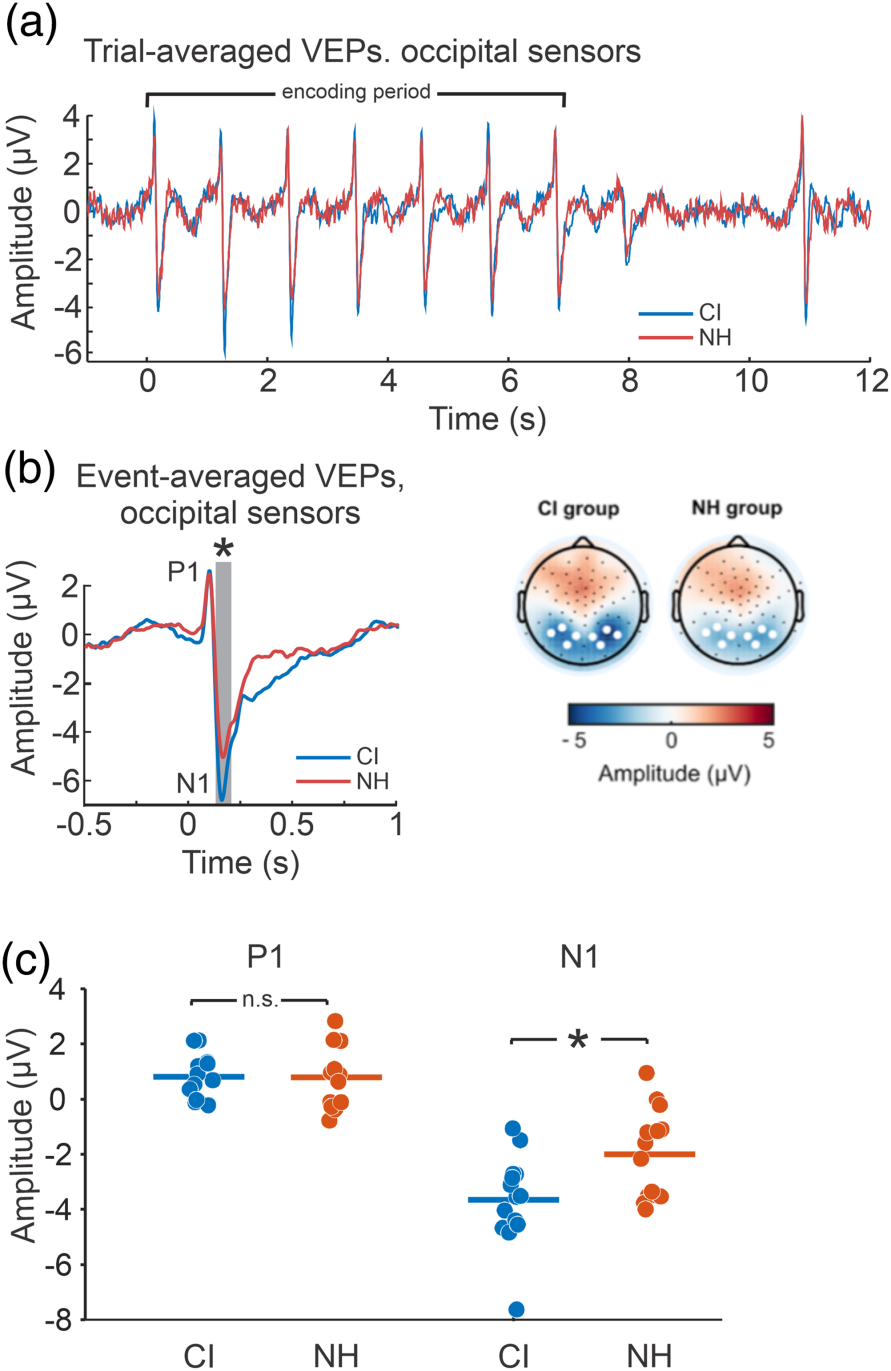
Cochlear implant (CI) users have larger visual N1 amplitudes; the difference is largest in right auditory cortical sources. (a) Voltage time series of visual‐evoked potentials (VEPs) over the trial for both groups, taken from occipital sensors shown in panel (b). (b) Comparison of normal‐hearing (NH) and CI VEPs averaged at each character onset (left panel) at occipital sensors highlighted as white dots on the topographical maps (right panel). The shaded region of the voltage time series in the left panel shows the significant difference between CI and NH groups. (c) Comparison of individual P1 and N1 values averaged across a 20‐ms time window around the peak P1 and N1 values. ^*^ *p* < 0.05

Trial‐averaged VEPs for each group over occipital electrodes are plotted in Figure 4a and show seven distinct P1–N1 complexes during the encoding period. An additional VEP‐like component was observed at 8‐s posttrial onset and may be related to a visual offset response. This latter response will not be considered further. The seven encoding VEPs were averaged together to produce one event‐related‐averaged VEP across eight posterior electrodes representing each subject's mean response to the presentation of a character (Figure 4b). P1s peaked at 0.096 and 0.092 s for the CI and NH groups, respectively, and N1s occurred at 0.156 and 0.160 s. An unpaired *t* test was conducted on an averaged 20‐ms window centred around P1 and N1 separately and revealed that P1 did not differ between NH and CI (*p* = 0.96) whereas the N1 was significantly greater in magnitude in the CI group compared with the NH group (*t*(26) = 2.69, *p* = 0.012, *η* ^2^ = 0.22).

#### 3.2.2 VEP source analysis differences between CI users and NH controls

**FIGURE 5:**
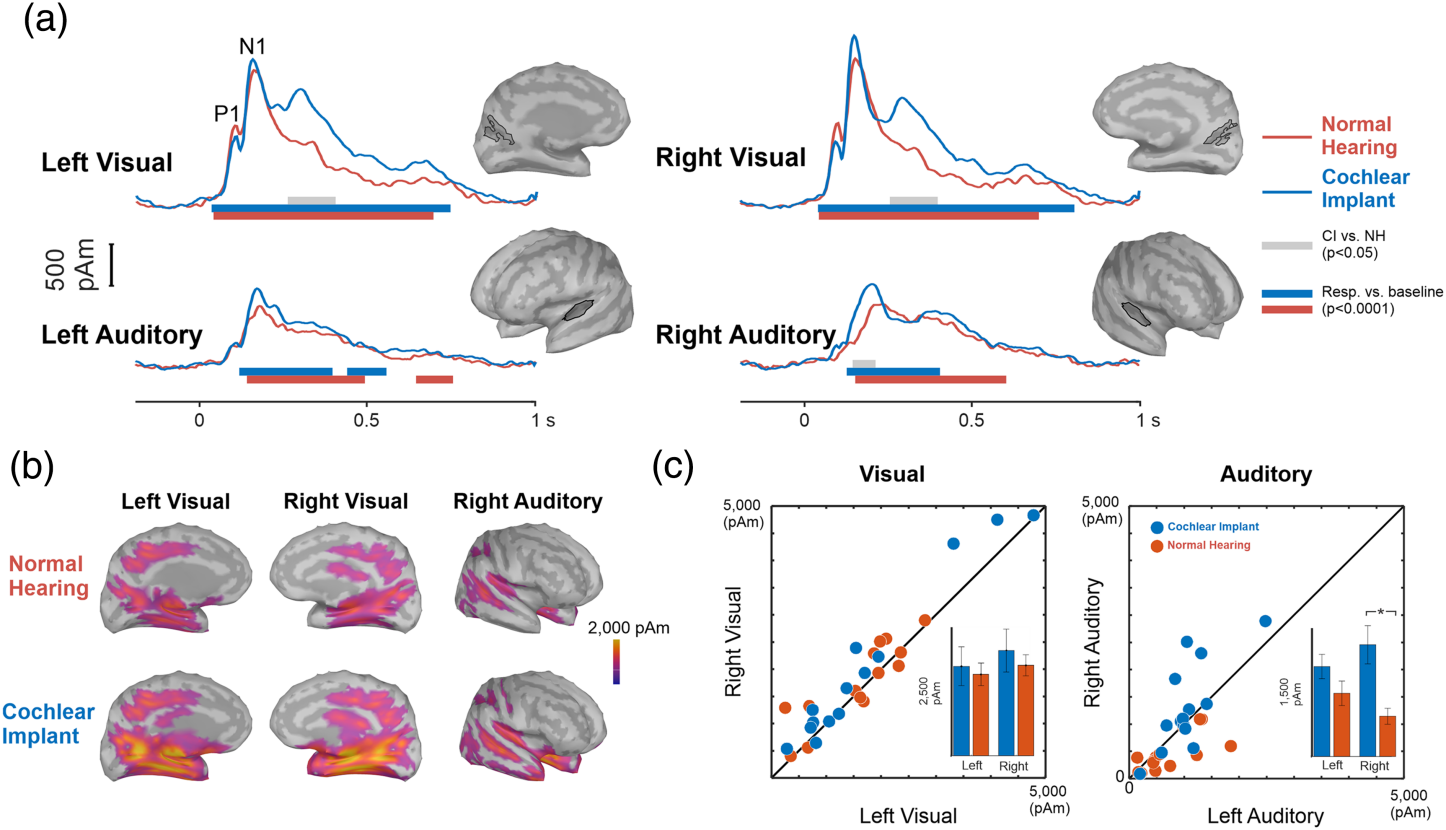
Event‐averaged auditory and visual activations during stimulus encoding in cochlear implant (CI) users and normal‐hearing (NH) controls. (a) Visual regions of interest (ROIs) displayed a peak corresponding to P1 and N1. In CI users, an additional peak occurred after the N1 response. Auditory ROIs only showed N1 response peaks. Also, CI users showed a delayed second peak in the right hemisphere. Grey horizontal bars indicate significant time points of differences between NH and CI, whereas red and blue bars indicate significant differences from baseline for NH and CI, respectively. ROIs are indicated by the black line on the cortical surface to right of each plot. (a) Whole brain standardized low‐resolution electromagnetic tomography (sLORETA) activations for the N1 time point. (c) Scatterplots showing individual N1 responses from the visual and auditory ROIs

ROI source time course activations were estimated using sLORETA in Brainstorm on the single event‐related‐averaged VEP (Figure 5). Multiple generators were observed for the P1/N1 response. The dominant generators were primary and secondary visual cortices, in addition to an anterior temporal parietal source and auditory cortex source. The activation time courses and spatial distribution were initially visually inspected and revealed that the secondary visual cortex was the dominant source followed by the temporal parietal source, then the primary visual cortex and finally the auditory source. We chose the secondary visual cortex as the ‘visual ROI’ because it was greatest in magnitude. Figure S1 summarizes all the ROIs. Differences between ROI activations across time (NH vs. CI) were initially assessed with unpaired *t* tests (as implemented in Brainstorm statistics) and were corrected for multiple comparisons using cluster‐based permutation testing. The visual ROI showed specific peaks corresponding to the P1 and N1 as seen in the sensor data. For the visual ROI, no differences between NH and CI groups were observed in time regions corresponding to P1 or N1 peak latencies. Cluster‐based permutation tests revealed that CI users had a significant difference in the right auditory N1 peak (*p* < 0.05). Descriptively, this cluster may be related to a difference in the N1 peak approximately from 160 to 190 ms, where the peak was larger in CI users compared with controls (Figure 5a).

Mean activations over a 20‐ms time window centred around P1/N1 peaks of the ROI activation was subjected to a 2 × 2 × 2 ANOVA (left/right × CI/NH × auditory/visual). For the N1, the ANOVA revealed a significant main effect for auditory/visual cortices such that visual responses were greater than the auditory responses (*F*(1,26) = 23.63, *p* < 0.001, *η* ^2^ = 0.21), in addition to a left/right auditory/visual interaction (*F*(1,26) = 6.603, *p* = 0.021, *η* ^2^ = 0.005) such that the right visual ROI was greater than the left visual ROI (*p* = 0.004). A CI/NH × left/right interaction was observed (*F*(1,26) = 7.40, *p* = 0.011, *η* ^2^ = 0.01) where CI users had larger right‐sided responses compared with NH controls. The three‐way interaction between CI/NH, left/right and auditory/visual was marginally significant (*F*(1,26) = 3.75, *p* = 0.064, *η* ^2^ = 0.003). Post hoc tests indicated that CI users had larger responses in the right auditory ROI (*p* = 0.018, Figure 5c), while no differences were found between the left auditory ROI or the visual ROIs (*p*s > 0.37). It should be noted that although the visual ROI (secondary visual cortex) did not differ between NH and CI groups in the N1 time range, significant differences between CI and NH were observed for the primary visual cortex ROI activations (*p* < 0.05) and appeared to relate to a cluster ranging from ~160 to 230 ms (unpaired *t* tests corrected for multiple comparisons performed in Brainstorm statistics; Figure S1).

A second visual ROI peak (296 ms) was observed in the CI group that was significantly different from the NH group (*p* < 0.05, unpaired *t* test corrected for multiple comparisons performed in Brainstorm statistics; Figure 5a). This cluster appeared to relate to the ~250‐ to 430‐ms period where CI users had larger activation magnitudes. The auditory ROI time series showed only a single peak in the N1 range. A 2 × 2 ANOVA comparing CI/NH and left/right secondary visual cortex indicated a significant interaction between group and visual ROI side (*F*(1,26) = 31.58, *p* < 0.001, *η* ^2^ = 0.28). Follow‐up tests indicated that CI users had a larger activation magnitude in the left visual ROI (*M* = 148.6, SE = 26.97) than NH controls (*M* = 84.6, SE = 16.73; *p* = 0.045). The difference for the right visual ROI was marginally significant (*p* = 0.07).

### 3.3 Differences in visually evoked oscillations between CI users and NH controls

**FIGURE 6:**
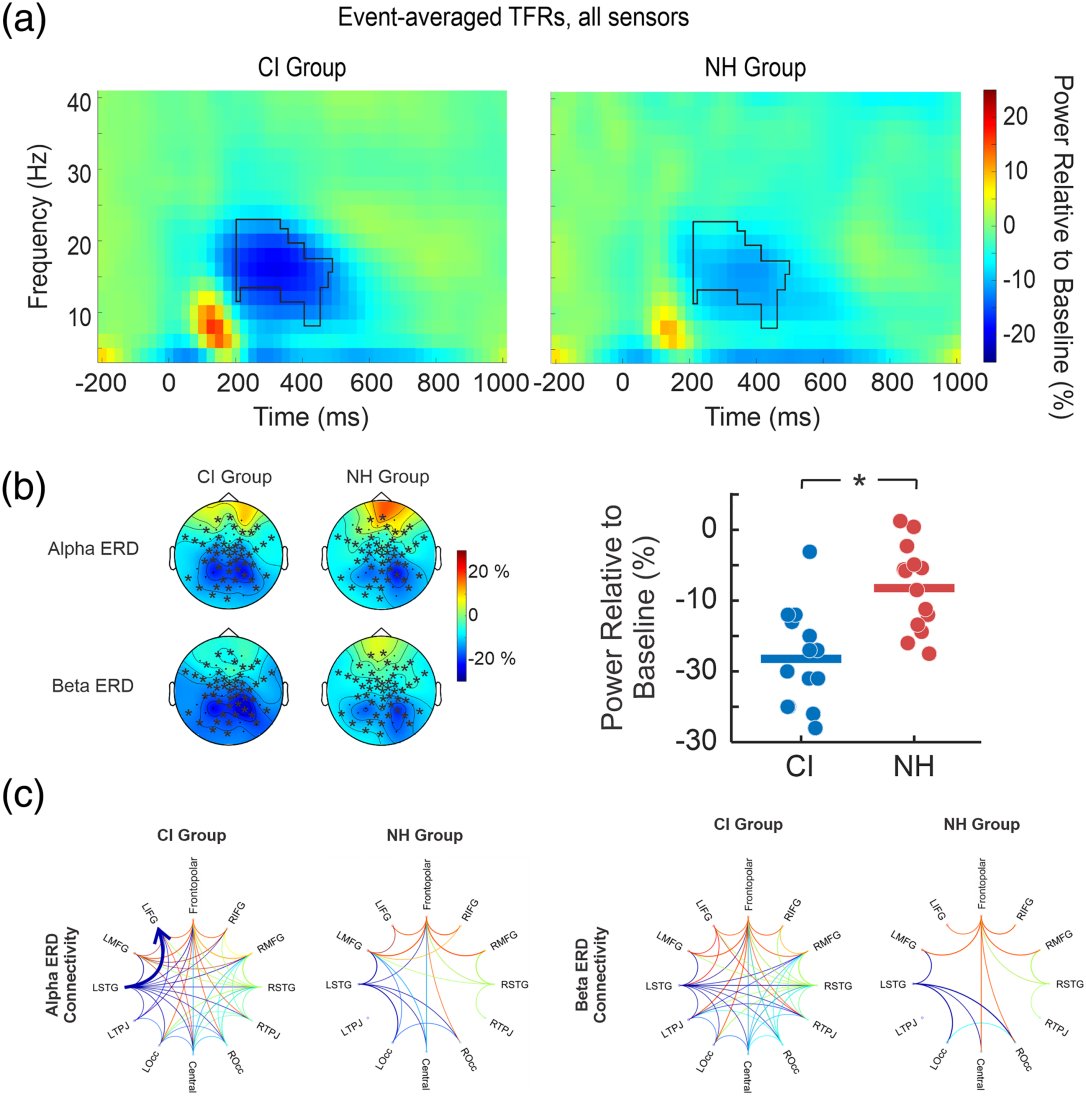
Comparison of alpha and beta event‐related desynchronization (ERD) during encoding. (a) Time–frequency representations (TFRs) for cochlear implant (CI) (left panel) and normal‐hearing (NH) groups (right panel) averaged at each character and across all sensors; significant differences in the alpha/beta ERD are outlined in black. Early portion of cluster (200–400 ms) includes higher alpha and beta components (11–22 Hz), and the latter portion (400–500 ms) includes lower alpha and beta (8–17 Hz). (b) Topography during the significant ERD portion (200–500 ms) with alpha (8–12 Hz) and beta (13–22 Hz) bands separated. (c) Comparison of connections during the alpha and beta ERD time and frequency windows for each group. Each coloured line represents the node in which the connection originates. All connections plotted are above a Granger causality (GC) value of 0.025. Individual connectivity strengths from the significantly different connections. The thick blue arrow indicates significant alpha ERD connectivity from left superior temporal gyrus (LSTG) to left inferior frontal gyrus (LIFG; *p* < 0.05) in the CI group. ^*^ *p* < 0.05

Figure 6a shows the grand averaged TFR for visually evoked oscillations after a character was presented (this was accomplished by averaging the TFRs of all seven characters) for each group across all channels. The late evoked potential observed in source space from ~250 to ~430 ms that was significantly different between the CI and NH groups appeared to overlap with a decrease in oscillatory power (event‐related desynchronization [ERD]) that occurred between 200 and 500 ms from 8 to 22 Hz (alpha/beta range). To test for frequency‐based differences, a cluster‐based test during the alpha/beta ERD suggested a significant difference between CI users and NH controls (*p* = 0.03) spanning a large number of electrodes centred on the occipital sensors (Figure 6b). Descriptively, the cluster spanned from 200 to 400 ms and overlapped with the beta frequency range (11–22 Hz) while the latter portion of the cluster (400–500 ms) decreases in frequency toward the alpha range (8–17 Hz).

#### 3.3.1 Differences in GC between CI users and NH controls

Following the significant cluster of group differences in the alpha and beta frequencies for late‐latency event‐related oscillations (Figure 6a), GC was calculated separately for the alpha and beta bands during the 200‐ to 500‐ms time range. Connections between each node are expressed as a GC value based on strength (higher values indicate stronger connections). For the alpha band, overall, connectivity during both time–frequency windows descriptively showed stronger connectivity across nodes in CI users compared with NH. Figure 6c shows the degree of alpha and beta frequency connections above the GC value threshold of 0.025 and are represented as lines between nodes.

Cluster‐based permutation tests correcting for comparisons conducted spatially across node connections indicated a significant difference between the NH and CI groups for alpha oscillations (*p* = 0.048). The result appeared to be driven by higher GC values directed from LSTG to LIFG in CI users compared with the NH group. For the beta band, no significant differences were found. A 2 × 2 mixed ANOVA on GC values directed from LSTG to LIFG for alpha and beta bands across groups indicated a significant interaction between frequency band and group (*F*(1,26) = 8.17, *p* = 0.008, *η* ^2^ = 0.035). Post hoc comparisons suggested that CI users had higher GC values in the alpha band directed from LSTG to LIFG compared with NH individuals (*p* = 0.0012), while no difference were found for the beta band (*p* = 0.12). Because LSTG and LIFG together are typically associated with speech and language processing, this finding suggests a higher communication between these areas during visual stimulus encoding in CI users in the alpha frequency band.

### 3.4 Retention period results

#### 3.4.1 Differences in TFRs between CI users and NH controls

**FIGURE 7:**
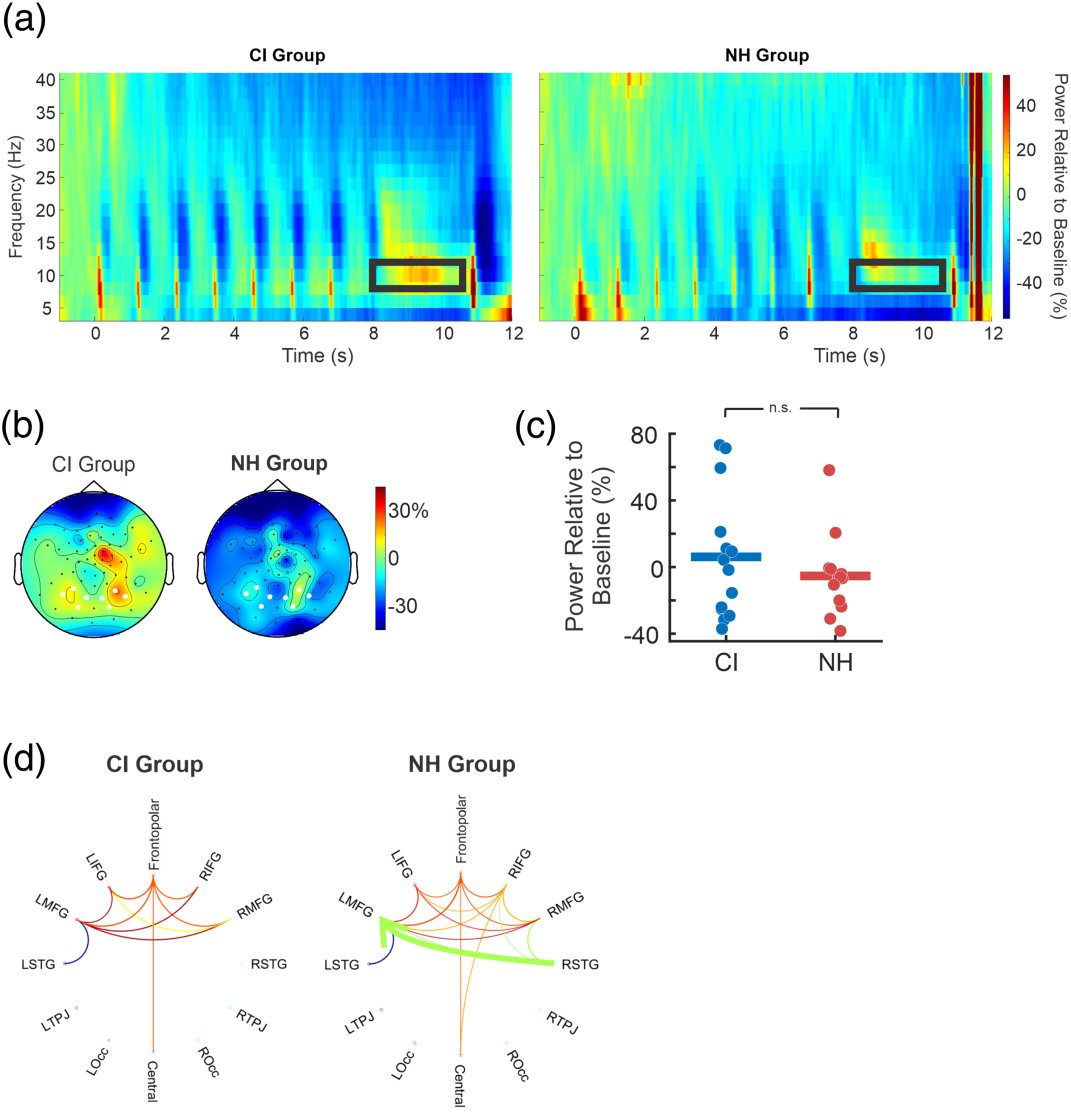
Comparison of alpha synchronization between normal‐hearing (NH) and cochlear implant (CI) users. (a) Time–frequency responses for CI (left panel) and NH groups (right panel) averaging the entire trial and across occipital sensors. Most significantly different areas highlighted by black box. (b) Topography during the retention alpha event‐related synchronization (ERS) and occipital sensors used are indicated by the white circles. (c) Scatter plot of individual alpha activations across the occipital channels during the retention period for both groups. (d) Alpha connectivity comparison of all connections during retention with each coloured line representing the node in which the connection originates. All connections plotted are above a Granger causality (GC) value of 0.025. Individual connectivity strengths from the significantly different connections. The thick green arrow indicates significant connectivity from right superior temporal guys (RSTG) to left medial frontal gyrus (LMFG; *p* < 0.05)

The grand average TFR in occipital channels across the entire trial is plotted in Figure 7. Observed are seven bursts of event‐related synchronization (ERS) and ERD around the onset of visual characters (the average of these events is shown in Figure 7a). Following these bursts, oscillatory activity in the retention period (8 to 10.5 s) appeared to differ between groups in the alpha band. Based on the group grand average, CI users had a delayed and long alpha ERS, while NH individuals had an earlier, short burst. Topographic maps of alpha power in Figure 7b suggest that alpha power is slightly right lateralized and distributed across occipital, parietal and central sensors. Inspection of individual data as an average of alpha power across the retention period however indicated that the grand average observations are skewed by a small number of participants in each group (Figure 7c), where the majority of individuals exhibit a pattern of ERD, not ERS. Thus, the pattern of differences appears to suggest more ERD in the NH group and less ERD in the CI group. Despite this qualitative difference, cluster‐based permutation tests of a group difference correcting across the entire sensory array but averaging across time points (8–10.5 s) and alpha frequencies (8–12 Hz) were not significant (*p* = 0.11).

#### 3.4.2 Source differences during retention between CI users and NH controls

As a further test for the hypothesis that alpha power differs between CI users and NH controls during the working memory retention period, a cluster‐based permutation test on the alpha source estimates resulted in a close, however, non‐significant difference (*p* = 0.086) driven by stronger ERD in the NH group in the occipital cortices peaking at Talairach (TAL): [−3.5, −93.9, −25.3] and in the parahippocampal regions peaking at TAL: [−17.5, −2.9, −25.3]. The results are shown in Figure S2. Across the whole brain, alpha ERD appears to be distributed frontally and ventrally in CI users, while the distribution in NH users spreads more occipitally. An exploratory breakdown of this finding was done by performing two separate cluster‐based tests centred on the apparent group average ERS for the NH group in the upper alpha to beta frequency range (11–16 Hz and 8.35–8.85 s) and the later difference in the alpha band for the CI group (8–12 Hz and 8.85–9.90 s), both peaking in the occipital area. Yet still, both subperiods were not significantly different (*p* = 0.15 and *p* = 0.09, respectively).

Considering both sensor and source results, the configuration of oscillatory alpha power differences between CI and NH groups only trended, perhaps due to a conservative correction for cluster formation across the entire sensor array and voxel space. For this reason, the marginal difference can be interpreted as having qualitative importance and appeared as a weaker desynchronization of alpha oscillations in occipital regions of CI users as the visual characters were held in working memory.

#### 3.4.3 Differences in GC between CI users and NH controls in the retention period

As a final test for the hypothesis that alpha oscillations differ between groups during the retention period, GC values were tested across brain nodes between NH and CI groups. Qualitative differences plotted in Figure 6e are suggestive of stronger connectivity patterns in frontal brain areas rather than in occipital regions. In contrast to connectivity results during the encoding of visual characters (Figure 6d), CI users had weaker patterns of connectivity compared with the NH group during retention (Figure 7d). Cluster‐based permutation tests for node connections indicated a significant group difference (*p* = 0.024). This result appeared to be driven by lower GC values in the CI group from RSTG to LMFG.

Taken together with alpha power differences, CI users generally appeared to have a less robust pattern of neural alpha oscillations while maintaining visual characters in working memory: the power of oscillations in occipital regions was qualitatively lower, and frontotemporal connectivity was significantly lower. The set of results during the retention period is a noteworthy reversal to larger evoked responses and stronger frontotemporal connectivity observed in CI users when visual characters were encoded prior to retention.

### 3.5 Brain–behaviour correlations

Correlation matrices were computed between all neural variables and task performance and subjectively rated effort and difficulty for both NH and CI groups. When assessed as isolated Spearman correlations, the relationship between difficulty and left and right P1 visual ROI activations (*ρ* = 0.61, *p* = 0.0207 and 0.68, *p* = 0.0072 respectively) were initially significant but did not survive significance after correcting for multiple comparisons.

For the CI group, correlations were computed between neural variables, speech‐in‐noise perception, age, duration of deafness and duration of CI use. The relationship between speech perception and all ROIs showed no correlation to the left and right visual activations (*ρ* = −0.08 and −0.04, respectively). The left and right auditory ROIs showed larger inverse correlations to speech‐in‐noise perception (*ρ* = −0.42 and −0.52, respectively) where the correlation with the right auditory ROI activation was trending but did not reach statistical significance (*p* = 0.07, uncorrected). No other correlations were significant.

## 4 DISCUSSION

### 4.1 Summary

This study was performed to investigate the neural differences in visual stimulus encoding and verbal working memory between CI users and age‐matched controls and how these differences may explain variability in CI users' speech‐in‐noise perception. A summary of findings is contextualized in terms of stated hypotheses. Inconsistent with Hypothesis (1), behavioural working memory performance was not different between CI users and NH controls. Hypothesis (2) was supported as CI users had larger visual N1 responses compared with NH controls, which appeared to be related to larger right auditory and visual cortex activation. In addition, a late potential from ~250 to ~430 ms indicated larger responses in CI users in secondary visual cortex. Event‐related alpha and beta oscillations decreased from baseline more strongly during visual character encoding, and connectivity was stronger between LSTG and LIFG. Also consistent with Hypothesis (3) was that connectivity was weaker during working memory retention directed from RSTG to LMFG in CI users, and descriptively, neural oscillations were not as strongly desynchronized during retention. Hypothesis (4) was not supported, as no neural variables that differed between groups explained variability in behavioural performance or self‐report of effort or difficulty. Finally, Hypothesis (5) was not supported; although the strength of activation in right auditory cortex to visual characters correlated negatively to speech‐in‐noise performance (*ρ* = −0.52), the result did not reach statistical significance (*p* = 0.07). The general pattern of results suggests that CI users had stronger neural activity during visual stimulus encoding and weaker neural activity during visual verbal working memory.

### 4.2 Working memory task performance and perceived task demand

Evidence suggesting performance differences between visual verbal working memory tasks in postlingually deafened CI users compared with NH controls is mixed. For example, during reading span, digit span and object span tasks, CI users performed similarly to NH (Kramer et al., 2018; Moberly, Houston, et al., 2017; Moberly, Pisoni et al., 2017) and are consistent with the behavioural results reported here. However, another study found poorer scores for CI users during a picture span task (Moberly et al., 2016). Perhaps this task was cognitively more demanding than the other tasks, and therefore, performance for CI users was hindered. In contrast, CI users outperform NH controls in performance during a symbol span task for which verbal labels are not used (Kramer et al., 2018; Moberly, Pisoni, et al., 2017), which can be taken as changes to visuospatial memory functions that could be related to an adaptive compensatory mechanism for navigating an environment. We can assume, considering the effort and difficulty scores in the present study, that both groups were comparable in how difficult they perceived the task to be and the amount of effort they believed to have allocated to complete the task.

### 4.3 Encoding of visual information in CI users

This study demonstrated that CI users had larger visually evoked N1 responses compared with NH controls during the encoding period of the trial. These observations generally agree with larger visually evoked P1, N1 and P2 responses evoked by sinusoidal concentric grating stimuli in early‐stage adult‐onset hearing loss (Campbell & Sharma, 2014) and the larger N1 and P2 amplitudes observed in deafness (Armstrong et al., 2002; Neville et al., 1983; Neville & Lawson, 1987). Some studies in contrast show smaller P1 amplitudes evoked by checkerboard patterns (Chen, Stropahl, et al., 2017; Sandmann et al., 2012), suggesting that visual plasticity has different effects depending on stimulus characteristics.

The increased N1 amplitude in CI users compared with NH controls suggests enhanced cortical activation in response to the visual stimuli. Source analysis suggested that the N1 activation was larger in CI users and appeared to be related to larger responses originating in auditory cortex. In addition, a late potential from secondary visual cortex was also higher in CI users compared with controls. The results can be taken as evidence of both cross‐modal and intramodal plasticity. However, we cannot conclude that auditory activations in CI users of the present study are driven by visual cortex activation nor are different from visual cortex activation. In general, activations of the auditory cortex by visual stimuli in CI users have been reported in the literature (Sandmann et al., 2012; Schierholz et al., 2015; Stropahl et al., 2015) and agreed with the view of visual reorganization of auditory brain areas. Enhanced visual cortex activity has also been previously reported in CI users (Doucet et al., 2006; Strelnikov et al., 2013). In contrast to these studies, we did not find that visual ROI activations were inversely correlated with auditory ROI activations, and the correlation between speech‐in‐noise ability and right auditory ROI activation did not reach statistical significance. Our findings do not replicate previous work showing that individuals with poorer speech perception have greater degrees of auditory reorganization (Buckley & Tobey, 2011; Sandmann et al., 2012; Schierholz et al., 2015), nor do they support the suggestion that there are cognitive consequences resulting from the reliance of visual stimuli before implantation such as impairments in memory and attention in that individuals with weaker speech perception might require increased cognitive resources to understand speech (Heald & Nusbaum, 2014).

One novel finding for visual character encoding was that an ERD in the alpha and beta frequency range was stronger for CI users compared with NH controls. This activity overlapped with the late cortical potential difference observed in visual cortex, but we cannot conclude whether or not these two differences originate from the same generators or neural processes. One view is that alpha oscillations represent active inhibition of sensory and task‐irrelevant information (Bonnefond & Jensen, 2012; Jensen & Mazaheri, 2010) and this may also be implied in certain contexts when alpha and beta power changes co‐occur (Händel et al., 2011; Kelly et al., 2006; Sauseng et al., 2009; Worden et al., 2000). Increases in posterior alpha ERD power during encoding have been shown to occur during visual N‐back tasks as cognitive load increases (Dong et al., 2015; Krause et al., 2000; Scharinger et al., 2015) suggesting that in higher demanding working memory tasks, posterior areas are implicated in operations that continually update and maintain information. This occurs, especially, in individuals with shorter working memory spans (Dong et al., 2015; Krause et al., 2000; Scharinger et al., 2015) and individuals with a lower intelligence quotient (Grabner et al., 2004). Based on these past results, the CI users' larger occipital alpha and beta ERD in this study may indicate a greater deployment of cognitive resources when encoding the visual characters, but notably, the correlation between demand and ERD was not significant, and the elevation in self‐reported task difficulty in CI users (which may reflect application of cognitive resources) did not reach statistical significance compared with NH controls. Thus, this assumption requires further testing.

A second novel result was that GC analysis during visual character encoding indicated significantly stronger connectivity in the alpha band from the LSTG to the LIFG in CI users compared with NH which, however, does not correlate to any behavioural measures. Previous studies show modified functional connectivity during auditory processing in CI users and individuals with hearing loss and greater recruitment of frontal areas in individuals with hearing loss during auditory perception (Chen, Puschmann, et al., 2017; Puschmann & Thiel, 2017). The STG, associated with auditory processing, and the IFG, associated with cognitive processes such as attention and working memory, have shown to be activated when attempting to comprehend degraded sentences (Davis et al., 2011; Wild et al., 2012; Zekveld et al., 2012) and are thought to be a part of a larger speech‐motor network (Hickok & Poeppel, 2007). These areas, along with the primary motor cortex, are also involved in the phonological loop for verbal rehearsal (Fegen et al., 2015; Herman et al., 2013), and activations were shown to increase as memory load increases (Fegen et al., 2015). The results of the current study do not seem to corroborate this finding; a possible explanation is that we did not alter memory load through the number of stimuli presented. However, the difference in connectivity between CI users and NH controls could be interpreted as a stronger dependency on speech networks or subvocal rehearsal for verbal memory storage in the former group.

### 4.4 Retention of visual information in CI users

Once all stimuli were presented, participants were required to hold the information in memory for 3 s before the probe was shown. In this study, CI users showed a trend of weaker alpha power compared with NH controls. A previous study investigated working memory by testing individuals with hearing loss on a modified auditory Sternberg working memory task in three levels of background noise and memory load (2, 4 and 6 digits; Petersen et al., 2015). The alpha oscillations, during retention, were higher in power in individuals with severe hearing loss under low and intermediate task demands (noise and memory load) compared with alpha power during the highest level of demand. Based on this study, individuals with hearing loss may have exceeded their ‘cognitive limit’ suggesting that in high demand tasks, resources are expended early for speech understanding leaving fewer to support the maintenance of information in a mental workspace.

This is also shown in NH groups; as load increases, alpha power decreases (Bashivan et al., 2014; Harmony et al., 1996; Stephane et al., 2008) suggesting that weaker alpha power is linked to poor maintenance and lower working memory capacity (Bashivan et al., 2014). However, alpha activity and the strength of it in working memory tasks is controversial; while some studies show decreases in alpha power, others show increases as the memory load increases (Hu et al., 2019; Jensen & Tesche, 2002) suggesting processing inhibition for competing stimuli. The general pattern of results in the present study suggests that the majority of individuals had a decrease in alpha power during retention compared with baseline, perhaps because cognitive load was not varied and the response task was to classify a target character and not fully reproduce the stimulus sequence. It is instructive to note that Pavlov and Kochoubey's review study, investigating the theta, alpha and gamma activities observed over 100 EEG and MEG studies, concludes that after comparison of the presentation of stimuli, modality and individual differences, there is no clear explanation for interstudy differences in alpha power (Pavlov & Kochoubey, 2020). Results may be better explained in terms of the specific task demands.

Connectivity during the retention interval, however, was weaker between the RSTG to the LMFG in CI users and may reflect the flow of sensory information to frontal areas involved in maintenance and retrieval. As a part of the dorsolateral prefrontal cortex, MFG is known to communicate with the STG and is involved in auditory processing (Barbas, 1992; Chavis & Pandya, 1976; Pandya et al., 1969; Pandya & Kuypers, 1969; Petrides & Pandya, 1988). The MFG is involved in the active maintenance of verbal information during retention long delays (Braver et al., 1995; Cohen et al., 1996; Fegen et al., 2015), associated with the manipulation of information in working memory (Champod & Petrides, 2007, 2010; D'Esposito et al., 1999; Postle et al., 1999) along with attentional refreshing (Bor et al., 2003; Druzgal & D'Esposito, 2003; Rypma et al., 1999) and word retrieval (Binder et al., 2009; Heim et al., 2009; Spalek & Thompson‐Schill, 2008; Whitney et al., 2009). The activation of the RSTG and its interhemispheric connectivity to the LMFG could be interpreted as an adaptation under increasing task demands, in which RSTG is recruited if the neural resources available in the language‐dominant LSTG are insufficient (Banich, 1998; Belger & Banich, 1998; Hellige, 1990). One study investigating the brain regions involved in audiovisual integration of letters showed that once the LSTG is activated for the auditory processing of visually presented letters, the RSTG is activated 70 ms later, suggesting communication between the two gyri via callosal connections (Raij et al., 2000). Thereafter, interhemispheric connectivity increases from RSTG to LSTG, which is similar to a finding showing increased connectivity from RSTG to LIFG finding observed under a rhyming judgement task (Bitan et al., 2010). If we apply this to CI users, this would provisionally suggest that CI users exhibit hemispheric differences when adapting to the cognitive demands of a certain task due to cross‐modal reorganization of right auditory cortex, which is unavailable to participate in bilateral processing of verbal stimuli.

### 4.5 Implications and future directions

This study shows several neural differences in encoding and maintaining visual verbal stimuli in working memory in CI users compared with NH controls, suggesting that CI users use comparatively more cognitive resources in the encoding of visual characters, as shown by the stronger evoked potentials, neural oscillations and frontotemporal connectivity and fewer resources during the retention interval as evidenced by weaker frontotemporal connectivity. Future studies should investigate both auditory and visual verbal memory systems and their neural correlates within the same participants, in order to compare overall memory function and how the balance of verbal memory changes as a function of sensory mode with restored deafness or hearing loss. Hearing loss, in previous studies, has been associated with impaired cognitive function and dementia (Lin, Ferrucci, et al., 2011; Lin, Metter, et al., 2011; Livingston et al., 2017; Lopes et al., 2007). It has been suggested that hearing loss places a larger demand on neural resources in order to process degraded auditory signals, leaving fewer resources for other cognitive processes such as language processing (Holtzer et al., 2009; Stern, 2009; Zarahn et al., 2007). This chronic reduction in resource availability may relate to the development of dementia later in life. There are, however, other factors that may account for the development of dementia or cognitive impairment associated with hearing loss, such as social isolation (Strawbridge et al., 2000) and sensory deafferentation itself (Lin, Ferrucci, et al., 2011). Clinical cognitive screening assessments have been developed, such as the Hearing‐Impaired Montreal Cognitive Assessment (HI‐MoCA), to determine the cognitive function of hard‐of‐hearing individuals before an aid is provided (Lin et al., 2017). This assessment may be a valuable tool to help explain the variability of CI performance after surgery.

### 4.6 Conclusion

Our investigation of encoding and retaining visual verbal information in working memory supports previous theories demonstrating both intramodal and cross‐modal plasticity in CI users (Doucet et al., 2006; Rouger et al., 2012; Sandmann et al., 2012; Strelnikov et al., 2013), and our findings complement prior reports showing altered cortical connectivity in CI users (Chen et al., 2017; Smieja et al., 2020). Neural correlates of visual character processing by way of VEPs, event‐related oscillations and frontotemporal connectivity were stronger in CI users over controls, but retention of verbal information in working memory assessed by neural oscillations and frontotemporal connectivity was weaker for CI users. The novel finding of great alpha and beta desynchronization suggests greater engagement of cognitive resources. Despite these differences, poor speech‐in‐noise outcomes for CI users did not significantly correlate to these neural changes and therefore do not strongly support previous theories of ‘maladaptive’ neural plasticity. The findings overall potentially clarify relationships between memory function and significant sensory loss that are of increasing interest due to the relationship between hearing decline and cognitive decline and dementia (Slade et al., 2020).

## CONFLICT OF INTEREST

The authors declare no conflicts of interest.

## AUTHOR CONTRIBUTIONS

PP designed the study, ran the experiment, drafted the paper and analysed the data. BTP designed the study and drafted the paper. TL designed the study and drafted the paper. JC designed the study and drafted the paper. VL designed the study and drafted the paper. AD designed the study, supervised the experiment, drafted the paper and analysed the data.

### PEER REVIEW

The peer review history for this article is available at https://publons.com/publon/10.1111/ejn.15365.

## Supporting information

**Figure S1.** Dominant generators associated with the visual P1/N1 responses. ROIs are indicated by the black outline on the cortical surface. The primary visual cortex did not yield well‐defined P1/N1 peaks and the responses were smallest of the three ROIs. The secondary visual cortex ROI was chosen as the dominant generator used for the manuscript since it had well defined peaks and was larger than the temporal parietal ROI. Significant time regions where CI activations are larger than NH are given. Note for temporal parietal ROI, the p‐value was 0.06 for a short time period in the left hemisphere.
**Figure S2.** Alpha source activation during the retention period. Comparison between CI users (left panel) and NH group (middle panel) with the difference shown as a red cluster (right panel) in the occipital cortex (top panel) and parahippocampal cortex (bottom panel). Crosshairs placed at the peak occipital cluster difference (TAL: [‐3.5‐93.9‐25.3]) and peak parahippocampal cluster difference (TAL: [‐17.5‐2.9‐25.3]). Note that these differences trended towards significance, but did not reach the 0.05 level; (p = 0.15 and p = 0.09 respectively).

## Data Availability

Data for this manuscript can be accessed from: https://figshare.com/authors/Andrew_Dimitrijevic/10980087.
