# Supplementary material for: Neural correlates of visual stimulus encoding and verbal working memory differ between cochlear implant users and normal‐hearing controls
Source: Eur J Neurosci. 2021 Jul 9;54(3):5016–37. doi: 10.1111/ejn.15365 (PMC8457219; doi:10.1111/ejn.15365)
Supplement: Supplementary file 1 — Figure S1. Dominant generators associated with the visual P1/N1 responses. ROIs are indicated by the black outline on the cortical surface. The primary visual cortex did not yield well‐defined P1/N1 peaks and the responses were smallest of the three ROIs. The secondary visual cortex ROI was chosen as the dominant generator used for the manuscript since it had well defined peaks and was larger than the temporal parietal ROI. Significant time regions where CI activations are larger than NH are given. Note for temporal parietal ROI, the p‐value was 0.06 for a short time period in the left hemisphere. Figure S2. Alpha source activation during the retention period. Comparison between CI users (left panel) and NH group (middle panel) with the difference shown as a red cluster (right panel) in the occipital cortex (top panel) and parahippocampal cortex (bottom panel). Crosshairs placed at the peak occipital cluster difference (TAL: [‐3.5‐93.9‐25.3]) and peak parahippocampal cluster difference (TAL: [‐17.5‐2.9‐25.3]). Note that these differences trended towards significance, but did not reach the 0.05 level; (p = 0.15 and p = 0.09 respectively). [file EJN-54-5016-s001.docx]

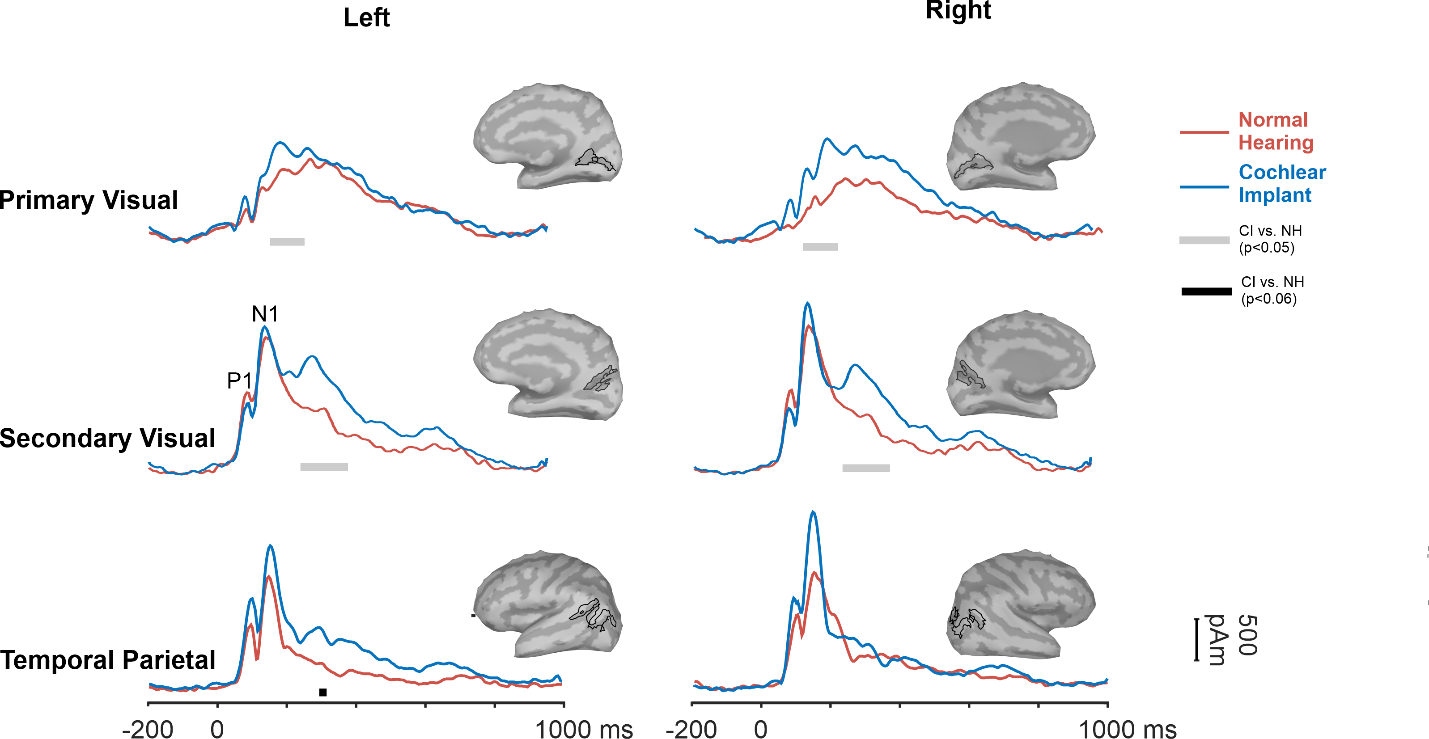


**Supplemental Figure 1.** Dominant generators associated with the visual P1/N1 responses. ROIs are indicated by the black outline on the cortical surface. The primary visual cortex did not yield well-defined P1/N1 peaks and the responses were smallest of the three ROIs. The secondary visual cortex ROI was chosen as the dominant generator used for the manuscript since it had well defined peaks and was larger than the temporal parietal ROI. Significant time regions where CI activations are larger than NH are given. Note for temporal parietal ROI, the p-value was 0.06 for a short time period in the left hemisphere.


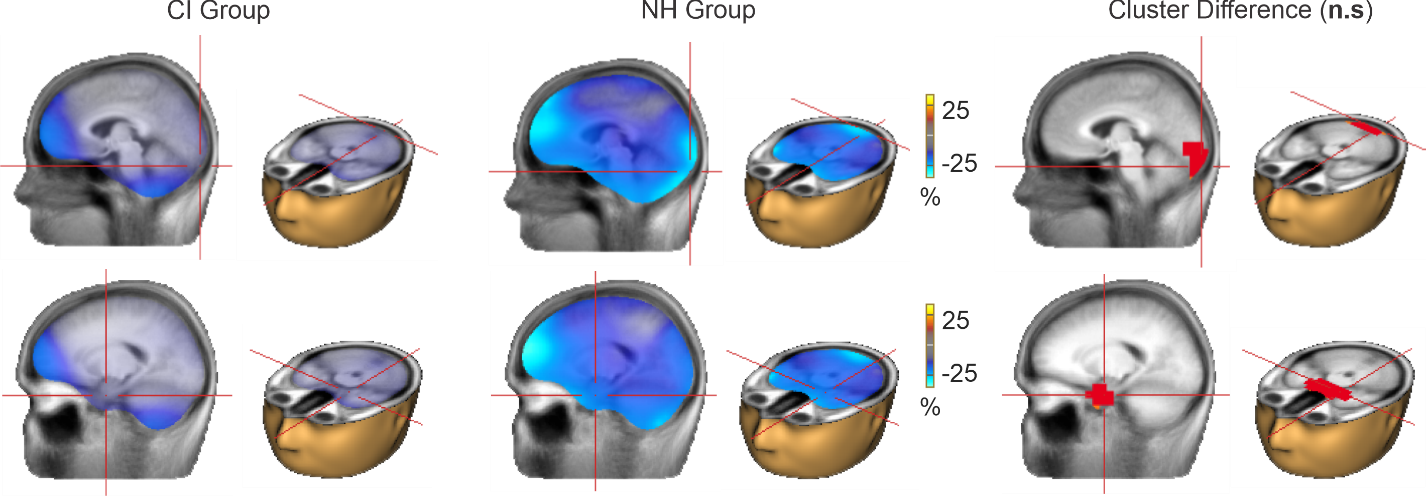


**Supplemental Figure 2.** Alpha source activation during the retention period. Comparison between CI users (left panel) and NH group (middle panel) with the difference shown as a red cluster (right panel) in the occipital cortex (top panel) and parahippocampal cortex (bottom panel). Crosshairs placed at the peak occipital cluster difference (TAL: [-3.5 -93.9 -25.3]) and peak parahippocampal cluster difference (TAL: [-17.5 -2.9 -25.3]). Note that these differences trended towards significance, but did not reach the 0.05 level; (p = 0.15 and p = 0.09 respectively).
